# Cryptococcal infection causing longitudinal extensive transverse myelitis in an immunocompetent individual: Case report and literature review

**DOI:** 10.3389/fneur.2023.1171572

**Published:** 2023-04-13

**Authors:** Ashwin Kumar Panda, Sourav Hazra, Aldrin Anthony, Suman Kushwaha

**Affiliations:** Institute of Human Behaviour and Allied Sciences, University of Delhi, New Delhi, India

**Keywords:** cryptococcus, LETM, longitudinally extensive transverse myelitis, immunocompetent adult, cryptococcal antigen

## Abstract

Cryptococcal CNS infections in immunocompetent individuals are occasionally reported in literature. The spinal manifestations of cryptococcal CNS infections are epidural abscess, chronic arachnoiditis, intramedullary granuloma, myelitis and vasculitis. We report a rare case of CNS cryptococcal infection presenting as a longitudinal extensive transverse myelitis (LETM) in an immunocompetent male. This report highlights cryptococcus as an important etiology among infectious causes in acute LETM patients in-spite of the immunocompetent status of the patient and the utility of CRAG (cryptococcal antigen) for diagnosis in such patients. We also present a literature review of all reported cases of cryptococcal myelitis.

## Introduction

Cryptococcus neoformans, is a well-known fungal infection in immunocompromised patients. The most common CNS presentations are meningitis, meningoencephalitis, cerebral parenchymal abscess [cryptococcomas], gelatinous pseudocyst and hydrocephalus. The spinal cord manifestations include epidural abscess, chronic arachnoiditis, intramedullary granuloma, myelitis, and vasculitis. These manifestations have been reported in immunocompetent patients (1, 2). Transverse myelitis as a presenting feature of CNS cryptococcal infection has also been reported in literature (3) but longitudinal extensive transverse myelitis has been rarely reported (4). Herein, we report a 48-year-old immunocompetent male presenting with LETM due to cryptococcal infection, who regained normal functional status following treatment. We present this case to highlight (1) Cryptococcus neoformans as an important differential of infectious causes of acute LETM even in immunocompetent individuals, (2) Utility of Cryptococcal Antigen (CRAG) for diagnosis of cryptococcal CNS infections in immunocompetent individuals. We also present a review of all reported cases of cryptococcal myelitis in literature.

## Case report

A 48-year-old male presented with a four-day history of acute onset progressive weakness of the lower limbs. The weakness started in the right lower limb and then progressed to involve the left lower limb leading to an inability to get out of the bed without support within 4 days of onset of symptoms. He also complained of sensory loss below the level of the upper abdomen with tingling and paresthesia in bilateral lower limbs. This was associated with a constant, deep-seated, ill-defined pain with dysthesias without any positional variation in the lower back and both lower limbs suggestive of a funicular pain. He also complained of urinary and stool retention. He had a history of fever with mild headache from 3 days prior to the onset of the weakness which persisted till admission (7 days). There were no significant past interventions, medical or family history. He belonged to the lower socio-economic strata and was a rickshaw puller by occupation. On examination, he was febrile and had a catheter *in situ*. He had hypotonia and motor weakness of lower limbs. The power was 2/5 in the right and 3/5 in the left lower limb, with absent deep tendon/superficial reflexes (anal/bulbo-cavernous) and mute plantar. He had brisk tendon reflexes in both the upper limbs. There was also complete loss of pain and temperature sensation below the T6 level. The sensorium, cognition, optic disc, cranial nerves, cerebellar system, and power in upper limbs were normal on examination. With this history and examination, the possibility of an acute transverse myelitis was considered.

**Figure 1:**
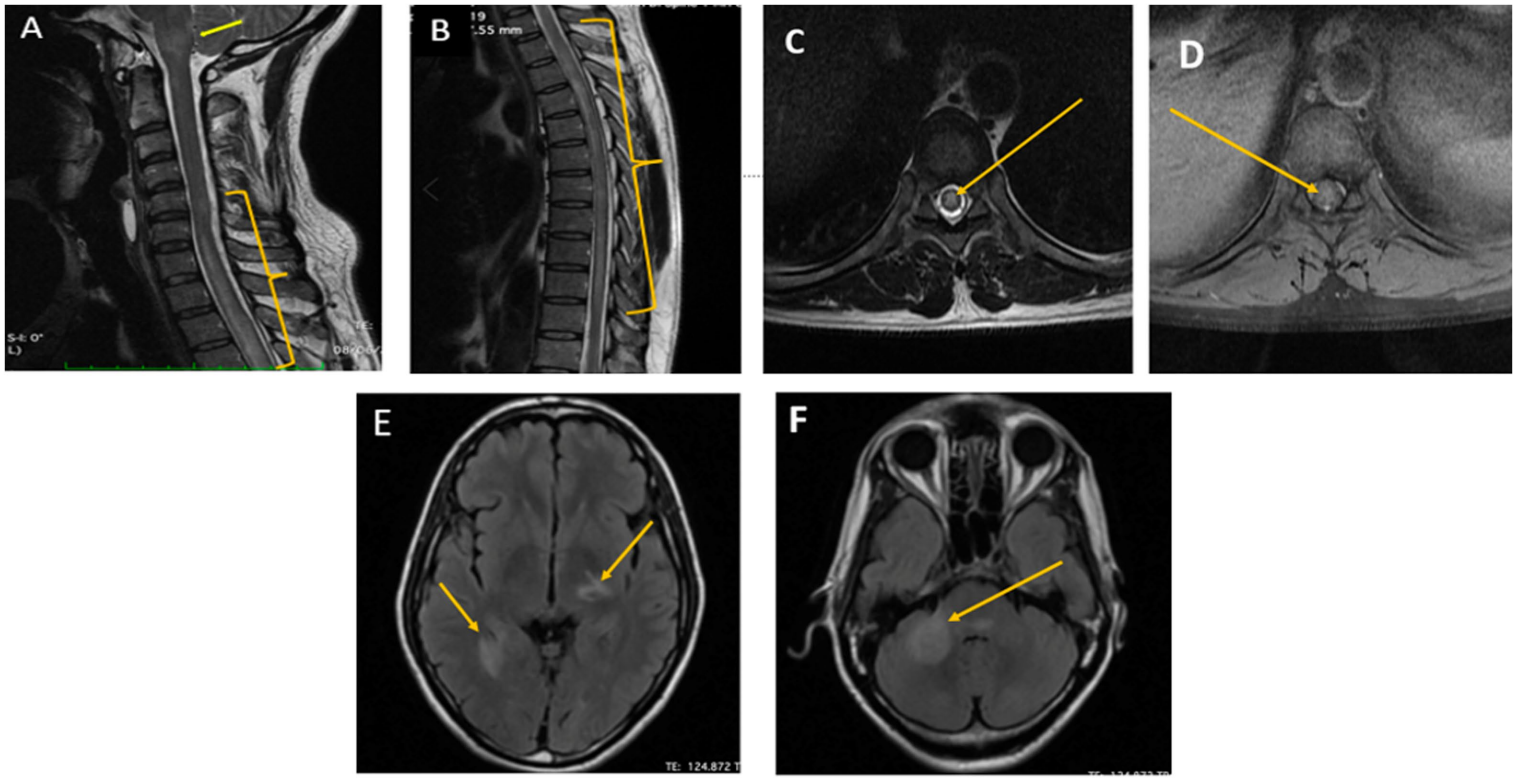
Pretreatment MRI cervicodorsal spine and brain image of the patient. **(A,B)** Sagittal T2/STIR images of the cervicodorsal MRI showed longitudinal extensive T2 hyperintensity in C5-D11 spinal cord segment (yellow bracket) along with brainstem involvement (yellow arrow). **(C,D)** Axial T2/STIR and T1 Contrast image of dorsal spine showing T2hyperintensity and partial enhancement, respectively, (Yellow arrow). **(E,F)** Axial FLAIR images how multiple hypointense lesions with surrounding hyperintensity in the left thalamus and the right mesial temporal and middle cerebellar peduncle suggestive of cryptococcal encephalitis (Yellow arrow).

The routine investigations including blood and urine cultures were negative. The contrast enhanced magnetic resonance imaging (CEMRI) of cervical-dorso-lumbar spine showed a T2W/STIR hyperintensity in the spinal cord extending from C7 to D11 vertebral level with partial enhancement, suggestive of a longitudinal extensive transverse myelitis (LETM). The CEMRI brain showed multiple T2W/FLAIR hyperintense lesions in the left frontal, right parietal and left temporal, periventricular white matter, pons, medulla and bilateral cerebellar lobes without any diffusion restriction or post contrast enhancement (Figure 1). Contrast enhanced MRI orbit did not reveal any significant abnormality. With these imaging findings, the diagnosis was revised to an encephalomyelitis. Infective, inflammatory, and demyelinating causes for LETM were investigated. Serum Antinuclear Antibody [ANA], Anti-Neutrophil Cytoplasmic Antibodies [ANCA] and Serum Neuromyelitis optica [NMO], Myelin oligodendrocyte glycoprotein [MOG] antibodies were negative. ELISA for serum viral markers of Hepatitis B, Hepatitis C and HIV were also negative. Serum Acetylcholinesterase (ACE) was normal [40 U/L]. Pathergy test was negative. Contrast enhanced computer tomography of chest and abdomen (CECT) did not reveal any abnormality. Visual evoked potential (VEP) showed bilateral normal P100 latency. The CSF examination showed decreased glucose, i.e., 67 mg/dl against the corresponding random blood sugar of 152 mg/dl and increased protein of 133 mg/dl with a cell count of 70 cells/mm3 (i.e., lymphocytes-56 cells/mm3, neutrophils-14 cells/mm3). No atypical cells were found. CSF gram stain, Ziehl-Neelsen [ZN] stain, India ink and CSF cultures were non-contributory. CSF neuroviral panel (Measles, Mumps, Epstein Barr, Parvo B19, Enterovirus, Varicella zoster, West Nile, Herpes simplex viruses) was also negative. CSF Cartridge based Nucleic Acid Amplification Test [CBNAAT] for Tuberculosis and Venereal Disease Research Laboratory [VDRL] was negative. However, CSF antigen was positive for Cryptococcus neoformans (CRAG) by lateral flow assay. Hence, a diagnosis of cryptococcal encephalomyelitis presenting as a LETM was made based on the clinical presentation and investigations. As the patient was immunocompetent and did not have any occupational exposure, detailed evaluation for any presence of cryptococcus in lungs/sinuses and CD4 counts for immunodeficiency were done but were found to be normal.

**Figure 2:**
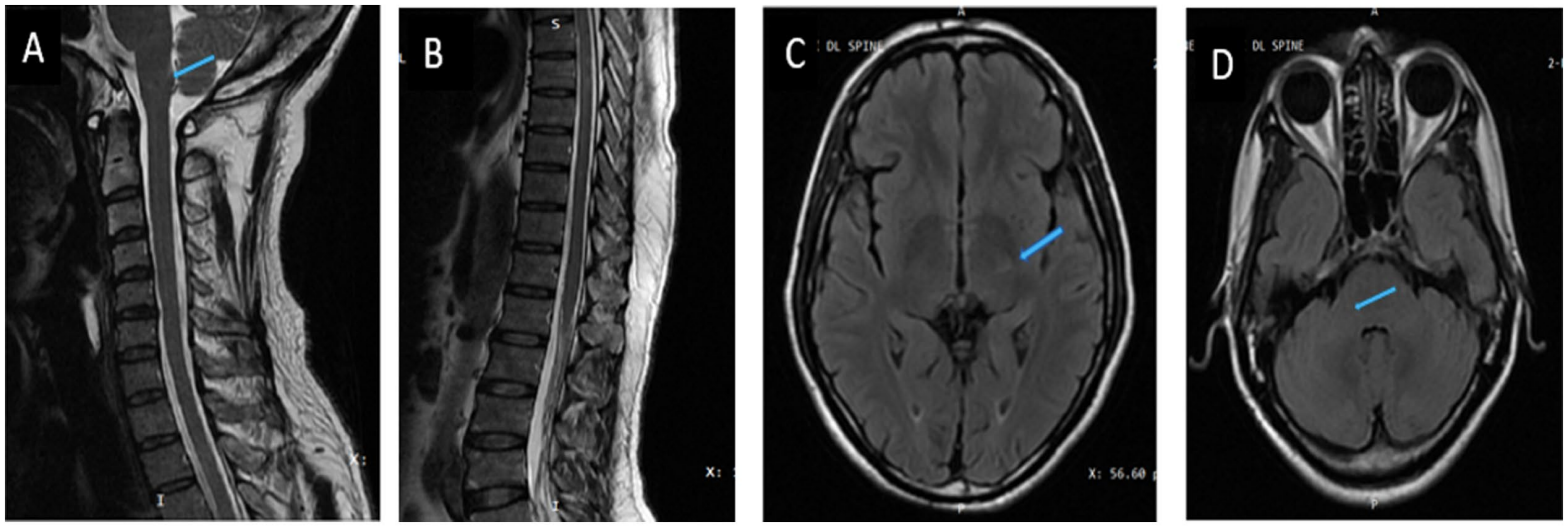
Post-treatment images (6 months after treatment). **(A,B)** Sagittal T2/STIR image of the cervicodoral MRI. **(C,D)** Axial T2FLAIR image of brain showing resolution of lesions (blue arrow).

**Figure 3:**
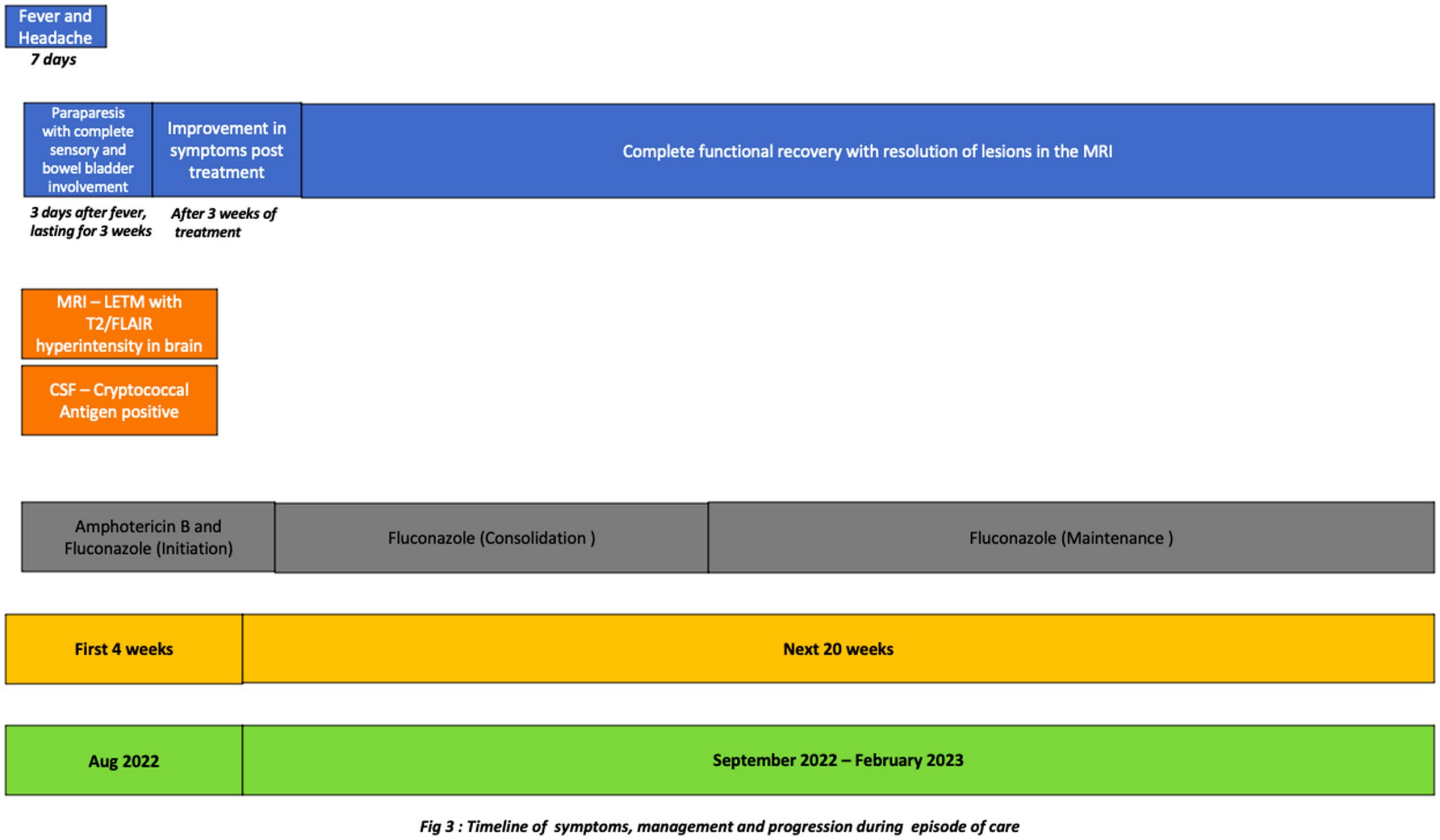
Timeline of symptoms, management and progression during episode of care.

The clinical team started the patient on 4 weeks of induction therapy with intravenous liposomal amphotericin B at 5 mg/kg [250 mg] every 24 h and oral fluconazole 200 mg every 8 hourly. After 3 weeks of antifungal therapy his motor power improved to 4/5 and 5/5 in the right and left lower limbs, respectively. His bladder and bowel symptoms resolved completely. His sensory loss resolved, although he had occasional paresthesia. He was discharged in a stable condition with an advice to continue oral fluconazole 400 mg/day for 12 months. The patient is in follow up, he had no side effects, tolerated the medicines and on subsequent examination has now regained full functional capacity. The follow up imaging done after 6 months showed near complete resolution of lesions (Figures 2, 3).

## Discussion

Our case presented a diagnostic challenge due to many unique features which we highlight in this report. (1) Cryptococcus presenting as an *acute LETM* in an *immunocompetent* patient, (2) Negative, Indian Ink for cryptococcus in the CSF, and (3) No source of cryptococcal infection found on investigations.

### Cryptococcus presenting as an acute LETM

LETM has been described in demyelinating, autoimmune, systemic vasculitis and infective conditions. Among infections, Herpesvirus (herpes simplex, varicella zoster virus, cytomegalovirus, Epstein–Barr virus), HIV, HTLV-1 are the commonly described viral causes. Bacteria such as treponema pallidum, mycobacterium tuberculosis, mycobacterium bovis, borrrelia burgdorferi and parasites like schistosomiasis have also been described as causative microorganism of LETM (5). Although other spinal cord manifestations of cryptococcus infections like epidural abscess, chronic arachnoiditis, both intra and extramedullary granuloma, myelitis and vasculitis have been described (6, 7). A cryptococcal infection causing an acute LETM has been rarely described in literature (8, 9).

We did a literature search (English language articles only) on Pubmed using the MeSH terms “myelitis” OR “transverse myelitis” OR “longitudinal extensive transverse myelitis” AND “cryptococcus” OR “cryptococcal infection” which yielded 13 cases (10 case reports) of paraparesis associated with cryptococcal infection. Of these, 5 cases had intramedullary cryptococcomas on imaging. Eight cases had myelitis, with 2 of them harboring intramedullary granulomas in addition to the myelitis. LETM was reported in only three cases (Table 1). Our patient is the fourth case of cryptococcal LETM in literature to the best of our knowledge. Our patient also had T2/ FlAIR hyperintensities on brain imaging in addition to the LETM without any alteration in sensorium. This was similar to a 44 year-old male reported by Villafuerte et al. who had presented with quadriparesis but had an abnormal MRI brain imaging (9).

**Table 1:** Clinical symptoms, imaging and management details of cryptococcal myelitis patients reported in literature.

Author/ Year/ Country	Age/Sex	Clinical features	Duration of symptoms	Primary infection site	Imaging findings	Lab features	Diagnosis	Immune status	Treatment	Outcome
Gumbo et al/1999-2000/Zimbabwe^(3)^	31/M	Left lower limb weakness, sensory loss and urinary incontinence; occipital headache	9 days	NA	MRI - Normal	CSF India ink-**positive** Cryptococcal antigen-**not done** C/S -**negative**	Encephalomyelitis	HIV+	Amphotericin B Oral fluconazole	Died
	31/F	Chest pain; Paraparesis	63 days	NA	CT D-L spine-Normal	CSF India ink and culture-**positive** Cryptococcal antigen- **NA**	Myelitis	Competent	Itraconazole	Normal 7months
	39/F	Paraparesis	13 days	NA	MRI D-L spine -Normal	CSF India ink and culture **Positive** Cryptococcal antigen-**NA**	Myelitis	HIV+	Amphotericin B Fluconazole	Normal 30days
Grosse et al/2001/Germany^(7)^	24/F	Paraparesis	90 days	Right lung	T2 hyperintense cranial and caudal to a ring like enhancement at L1 6 Ring enhancing lesions in the brain	Serum Cryptococcal antigen-**positive** CSF India ink-**negative** HPE c/s-**positive**	Encephalomyelitis	Competent	Amphotericin B + 5-flurocytosin + fluconazole+ operation	Normal 1year
Rai et al./2014/India^(8)^	18/M	Paraparesis	150 days	NA	MRI spine-long segment dorsal cord (D4-D13) patchy hyper intensity in T2	CSF antigen and India ink**positive**	LETM	Competent	Fluconazole	Normal 6months
	39/M	Paraparesis	90 days	NA	MRI spine-Normal	CSF antigen**positive**	Myelitis	Competent	Fluconazole	Normal 8months
Qu et al./2020/China^(4)^	55/M	Paraparesis	10 days	Lung	Longitudinal Extensive T2 hyperintensity (8 segments) and swollen thoracic cord with a Ring enhancing nodule at T9	Lung biopsy-**PAS staining positive** CSF Ag, India ink and culture-**negative**	Disseminated cryptococcosis	competent	Liposomal amphotericin B +Fluconazole+ Intrathecal dexamethasone	Normal 3months
Villafuerte/\2022/USA^(9)^	44/M	Headache Quadriparesis	5 days	NA	MRI Brain-Multifocal supratentorial ovoid and nonspecific T2 hyperintensities. LETM with cord expansion from cervico-medullary junction till C7	CSF antigen and culture**positive.** Confirmed by**PCR.**	Encephalomyeltis	competent	Liposomal amphotericin B +Flucytosine +Fluconazole	Normal Lost to follow up

CSF, Cerebrospinal fluid; C/S, Culture and sensitivity; CT, computer tomography; D/L, Dorso-lumbar spine; NA, Not available; MRI, Magnetic resonance imaging; PCR, polymerase chain reaction; PAS-Periodic acid schiff.

Overall, in cases of cryptococcal myelitis the median duration of onset of symptoms prior to admission was 38 days (IQR 9.5, 90). In the three cases of LETM reported in literature the duration of symptoms was 5, 10, and 150 days, respectively, (4, 8, 9). In our case, the patient had a four-day history of weakness prior to admission. This highlights that fungal diseases can also have acute CNS manifestations like a LETM.

### Immunocompetent status of the patient

Usually, cryptococcus infections are seen in immunocompromised individuals who either have HIV or have undergone solid organ transplant and are on immunosuppressive therapy. Other than these, patients with organ failure syndromes, innate immunologic problems, common variable immunodeficiency, and hematologic disorders are also reported with cryptococcal infections (10). Cryptococcus infections have been reported in immunocompetent individuals. Study in Australia, reported that 30% of total CNS cryptococcal infections occurred in immunocompetent individuals (11). It was also worth noting that, 75% (i.e., *n* = 6/8) of the cryptococcal myelitis cases reported in literature were immunocompetent individuals. In immunocompetent patients, robust CD4 T cells and Th1 inflammatory response causes inflammation, leading to clearing of the fungi and tissue damage. This is in contrast to a pauci-inflammatory and high infective burden state in the immunocompromised individual (12). Further, formation of granulomas is postulated to be influenced by an effective immune system through a trojan horse mechanism, i.e., passage across the cortical vasculature “within” phagocytes and neutrophils (13). Also, Liu J et al. mention a probable interaction of HLA class II alleles with cryptococcal meningitis, they report increased susceptibility and severe focal neurological deficits in patients with DQB1*05:02 loci (14). These mechanisms may explain the clinical presentation of our patient and the preponderance of immunocompetent individuals with myelitis in the literature.

### Diagnosis of cryptococcal infection

In our patient, cryptococcus infection was diagnosed by a positive CRAG (in CSF) by lateral flow assay. As a CNS infection was suspected (owing to the CSF picture), the antigen was sent despite a negative report of Indian Ink and culture for cryptococcus. Indian Ink is a rapid and easy method of diagnosing cryptococcosis but has a limitation in individuals with low fungal burden, i.e., non-HIV or immunocompetent individuals. Boulware et al. in their seminal paper mention that Indian Ink testing has a low sensitivity when compared to CRAG, this further decreases in patients with a CFU of <1,000/mL in CSF cultures. They also report that Indian Ink has a negative predictive value of only 80%. The sensitivity of Indian Ink in non-HIV patients varies from 30 to 72% whereas the sensitivity and specificity of CRAG is >99% (15). Our case emphasizes that when suspecting cryptococcus, CRAG is a better diagnostic modality in an immunocompetent individual than an Indian Ink test despite the latter’s cost effectiveness and wide availability.

### No source of cryptococcal infection

Our patient had a CNS cryptococcal infection without any evidence of a systemic source. In the literature review of similar cases of myelitis (Table 1), only 2 cases of myelitis had a source of infection in the lung. Further, in 62% of the cases of myelitis reported in literature (*n* = 5/8), cryptococcus neoformans was isolated (Table 1). But we could not identify the genotype or speciate the cryptococcal infection found in this case. LETM has only been reported in cryptococcus neoformans and no cases of C. Gatti have been reported yet although C. Gatti is more common in immunocompetent individuals. As, we did not do a typing, C. Gatti can still be a possibility in our case. It is postulated that various factors like the species/strains/genotype of cryptococcus, host immunity and exposure determine the pathogenicity and further manifestations, i.e., only CNS manifestation or disseminated infection with lung/sinus involvement (16). Hence, we may not find a source of dissemination in all patients of cryptococcal CNS infection. The patient in our case did not have any occupational exposure in terms of dealing with pigeons/birds or soil or trees/forests, all of which have been reported as possible routes of de-novo infection. This makes a compelling argument toward a long term latent infection in an individual later causing cryptococcal infections (17).

### Treatment and outcome

The patient in our case recovered from the LETM clinically within 4 weeks of the induction therapy. He was put on consolidation therapy and is currently on maintenance therapy and has completely recovered on follow up. Others’ studies (4, 8, 9) also showed that correct diagnosis and prompt treatment results in 90% of patients showing complete recovery. This highlights the notion that if there are clinical pointers toward an infective etiology in case of a LETM, one should rule out cryptococcus CNS infection as it is a treatable condition (18).

### Strengths and limitations

In this case we could not speciate the cryptococcus, so we were unable to link the species and genotype of cryptococcus with the clinical symptoms and course of the illness. Although, the literature review revealed cryptococcal neoformans as an agent in majority of cryptococcal LETM. This case follows the patient from the onset of symptoms to a complete recovery from them, iterating the importance of investigating for treatable/infectious conditions implicated in LETM, as gaining neurological functionality in such individuals is possible. This case also emphasizes the need to use CSF CRAG as a diagnostic modality in immunocompetent individuals as low fungal burden in such individuals leads to negative Indian Ink and culture reports.

### Conclusion

1. LETM is a rare but important manifestation of cryptococcus infection, and it should be considered as an important cause of acute LETM.
2. Cryptococcal Myelitis and LETM have been mostly reported in immunocompetent individuals.
3. In most cases of cryptococcal LETM, cryptococcus neoformans was isolated as the causative species.
4. Diagnosis by CRAG should be considered even if Indian Ink and culture is negative, especially in immunocompetent individuals.
5. No source of dissemination may be found in individuals with CNS cryptococcal infection.
6. Correct diagnosis and prompt management prevents the progression of symptoms and permanent disability.

## Data availability statement

The raw data supporting the conclusions of this article will be made available by the authors, without undue reservation.

## Ethics statement

Written informed consent was obtained from the individual(s) for the publication of any potentially identifiable images or data included in this article.

## Author contributions

AP and SK: conceptualizing drafting and editing the manuscript. SH and AA: diagnosis, management of the case, and first draft of the manuscript. All authors contributed to manuscript revision, read, and approved the submitted version.

## Conflict of interest

The authors declare that the research was conducted in the absence of any commercial or financial relationships that could be construed as a potential conflict of interest.

## Publisher’s note

All claims expressed in this article are solely those of the authors and do not necessarily represent those of their affiliated organizations, or those of the publisher, the editors and the reviewers. Any product that may be evaluated in this article, or claim that may be made by its manufacturer, is not guaranteed or endorsed by the publisher.
